# The Royal Alexandra Hospital for Children, Rhyl

**Published:** 1904-01-30

**Authors:** 


### THE ROYAL ALEXANDRA HOSPITAL FOR CHILDREN, RHYL.

THE LADY SUPERINTENDENT writes: May I add a few more particulars of our hospital to those in your article, January 16? The dispensary, kitchen, and offices, sea-water baths, sea-water swimming bath, light-room containing *x*-rays and Finsen lamp, men's accident ward with nurse's room adjoining, recreation-room, the same size as the wards, porter's room, splints-room, are in the basement—which is not shown in your plans.

"Isolation" ward is perhaps rather misleading, as it suggests infectious diseases. "Separation" ward for serious cases or dying patients would be more correct. The Isolation Hospital for infectious cases is quite distinct, and is two miles distant. There is a sisters' sitting-room on both ground and first floor, the bedrooms are on the second floor.

I beg to enclose a pamphlet, which, on page 9 and on, will give you fuller details of the building.

Please accept our thanks for giving an account of the building in THE HOSPITAL, as it has such a large circulation, and we are anxious that it should be well known.

